# Online Dynamic Nomogram for Predicting 90‐Day Prognosis of Patients With Primary Basal Ganglia Cerebral Hemorrhage After Microscopic Keyhole Craniotomy for Hematoma Removal

**DOI:** 10.1002/brb3.70344

**Published:** 2025-02-19

**Authors:** Hongliang Wang, Sai Li, Yang Nie, Chenxi Chang, Haoyuan Wu, Bing Zhao

**Affiliations:** ^1^ Department of Neurosurgery The Second Affiliated Hospital of Anhui Medical University Hefei People's Republic of China; ^2^ Cerebral Vascular Disease Research Center Anhui Medical University Hefei People's Republic of China

**Keywords:** microscopic keyhole craniotomy for hematoma removal, nomogram, predict, primary basal ganglia cerebral hemorrhage, prognosis

## Abstract

**Objective:**

Primary basal ganglia cerebral hemorrhage (PBGCH) is the most common type of hypertensive intracerebral hemorrhage. Microscopically removing the hematoma via keyhole or microbone window craniotomy remains the most common surgical method in many hospitals across China for treating cases of primary basal ganglia hemorrhage exceeding 30 mL. The aim of this study was to establish a new practical evaluation system based on preoperative clinical and imaging factors to predict the short‐term prognosis of PBGCH after microscopic keyhole craniotomy for hematoma removal (MKCHR), providing a reference for clinicians and patients' families in deciding whether to proceed with surgery.

**Methods:**

A retrospective analysis was performed on 74 cases of PBGCH treated with MKCHR. Patient prognosis was assessed at 90 days postsurgery using the modified Rankin Scale. This study employed R software to conduct both univariate and multivariate logistic regression analyses aimed at identifying preoperative factors that influence short‐term prognosis following MKCHR. Additionally, a web‐based interactive nomogram was developed to forecast outcomes for PBGCH patients receiving MKCHR treatment. Model robustness was gauged using the concordance index (*C*‐index) and receiver operating characteristic (ROC) curve. Internal validation involved bootstrap resampling and calibration. Clinical utility was assessed via decision curve analysis (DCA), clinical impact curve (CIC), and net reduction interventions (NRI).

**Results:**

Glasgow Coma Scale (GCS) score ≤ 6, hemorrhagic volume > 102 mL, brain herniation, age > 58 years (*p* < 0.05) were independent risk factors for poor prognosis after MKCHR. The online dynamic nomogram website is https://sjwkalg.shinyapps.io/DynNomapp/. The model's *C*‐index and area under the ROC are both 0.899 (95% confidence interval [CI], 0.817–0.980). Following 1000 bootstrap resamples, the calibration curve indicates that the dynamic nomogram's predicted values closely match the observed values.

The models of DCA, CIC, and NRI show good clinical application.

**Conclusion:**

The online dynamic nomogram developed in this study demonstrates high predictive accuracy. This platform is characterized by its noninvasive and convenient nature, which facilitates the formulation of clinical treatment strategies. It offers a reliable data reference for preoperative surgical decision‐making in patients with PBGCH, thereby aiming to achieve beneficial outcomes.

## Graphical abstract

In this study, we created the first online dynamic nomogram to predict the 90‐day prognosis of patients with primary basal ganglia cerebral hemorrhage following microscopic keyhole craniotomy for hematoma removal. This tool assists in surgical strategy selection and postoperative prognosis evaluation. A graphic abstract was created using Figdraw.com.

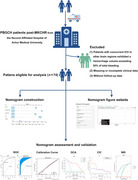

## 1 Introduction

Primary basal ganglia cerebral hemorrhage (PBGCH) is a form of cerebral hemorrhage characterized by high mortality and disability rates. It is the most common type of primary hypertensive cerebral hemorrhage, accounting for approximately 50%–70% of all cerebral hemorrhage cases (Li et al. 2017; Sutherland and Auer 2006). The disease manifests rapidly, characterized by the classic triad of symptoms: contralateral hemiplegia, sensory deficits, and homonymous hemianopia, among others. Substantial hemorrhaging may result in disturbances of consciousness and progression to coma. The prognosis is considerably poorer compared to that of cerebral lobe or cortical hemorrhages, with 70% of patients facing death or lifelong dependence (Kwon et al. 2018). Currently, there is ongoing debate among neurosurgeons worldwide regarding the surgical treatment of primary basal ganglia hemorrhages with volumes exceeding 30 mL (Morris et al. 2024). Previous major clinical trials have shown that conventional craniotomy to evacuate hematomas from the basal ganglia does not significantly improve functional outcomes for patients (Mendelow et al. 2005, 2013). However, with advancements in medical technology and instruments in recent years, minimally invasive surgical techniques have demonstrated potential in improving mortality and clinical outcomes associated with intracerebral hemorrhage (ICH; Lee et al. 2024; Pradilla et al. 2024; Shao et al. 2020; Sun et al. 2024). Representative procedures include small bone window or keyhole craniotomy for hematoma evacuation, endoscopic hematoma removal, and stereotactic‐assisted intracranial hematoma puncture and aspiration, among other methods. Among these, microscopic keyhole or microbone window craniotomy for hematoma has become a routine approach in many hospitals across China for treating cases of primary basal ganglia hemorrhage exceeding 30 mL, as it offers a life‐saving intervention.

Currently, neurosurgeons around the world are researching the risk factors associated with poor prognosis in patients experiencing cerebral hemorrhage. These factors include variables such as age, Glasgow Coma Scale (GCS) score, preoperative hematoma volume, hematoma location, vascular abnormalities, and hematological markers (Dinc et al. 2020; Hazra et al. 2023; Ji et al. 2013; Liu et al. 2020; Sun et al. 2019; Zhou et al. 2022). Several clinical prediction models have been developed to identify patients at high risk for unfavorable outcomes, such as the static assessment system created by the Zhou et al., which uses clinical and imaging features to predict the 7‐ to 10‐day postoperative prognosis for patients with basal ganglia cerebral hemorrhage (Zhou et al. 2022). Additionally, Lin et al. developed a model to predict long‐term survival in patients with spontaneous ICH (Lin et al. 2023). However, there is currently no scoring system or predictive model available to assess the short‐term prognosis of patients with PBGCH who are undergoing microscopic keyhole craniotomy for hematoma removal (MKCHR). Furthermore, existing prognostic predictions for cerebral hemorrhage primarily provide static evaluation systems, which are not particularly convenient for clinicians or patients. This research endeavors to establish an innovative and pragmatic predictive evaluation framework that synthesizes existing prognostic determinants influencing outcomes postminimally invasive surgery for PBGCH. By incorporating preoperative clinical and imaging variables, the study aims to evaluate the short‐term prognosis of patients undergoing MKCHR. The findings are anticipated to guide patient consultations and assist surgeons in surgical planning.

## 2 Materials and Methods

### 2.1 Patient Data

The clinical data of 74 patients diagnosed with PBGCH at the Second Affiliated Hospital of Anhui Medical University between August 2018 and June 2022 were analyzed retrospectively. All patients had no surgical contraindications and were deemed suitable for MKCHR. The characteristics of the study participants are presented in Table 1. This retrospective study received approval from the Ethics Committee of the Second Affiliated Hospital of Anhui Medical University (Approval Number: 20240102). Informed consent was obtained in writing from all patients. PBGCH is diagnosed according to the latest current guidelines for ICH: the American Heart Association/American Stroke Association Guidelines for the Management of Patients with Spontaneous Intracerebral Hemorrhage, Edition 2022 (Greenberg et al. 2022). All patients received a uniform clinical and radiographic assessment, including basic personal information, a retrospective analysis of symptoms and history, GCS scores, and multiple metrics from high‐resolution computed tomography (CT). The hematoma volume was calculated by multiplying the longest and widest diameters from the hemorrhage's maximum cross‐section in high‐resolution CT images, then multiplying by the hematoma's thickness (number of layers times layer thickness), and dividing by 2. Following thorough evaluations, the same team of experienced neurosurgeons determined and executed MKCHR. Additionally, patients presenting with concurrent ICH in other brain regions, where the hemorrhage volume exceeded 30% of the total volume of bleeding, were excluded from the study. Patients with missing or incomplete clinical data—including medical history, high‐resolution CT data on multiple indicators, or a brief period of clinical follow‐up—were also excluded.

**TABLE 1:** Characteristics of the 74 patients.

Characteristic	Value
Age (years); *n* (%)	
≤ 58	43 (58.11)
> 58	31 (41.89)
Gender, *n* (%)	
Male	53 (71.62)
Female	21 (28.38)
History of smoking or alcohol use, *n* (%)	
No	25 (33.78)
Yes	49 (66.22)
Junior high school and beyond, *n* (%)	
Yes	39 (52.70)
No	35 (47.30)
Residence, *n* (%)	
City	40 (54.05)
Countryside	34 (45.95)
Time from onset to admission (h); *n* (%)	
≤ 3 h	43 (58.11)
> 3 h	31 (41.89)
GCS score, *n* (%)	
> 6	59 (79.73)
≤ 6	15 (20.27)
History of ischemic stroke, *n* (%)	
No	39 (52.70)
Yes	35 (47.30)
Hyperglycemia, *n* (%)	
No	35 (47.30)
Yes	39 (52.70)
Hyperlipidemia, *n* (%)	
No	61 (82.43)
Yes	13 (17.57)
Regular intake of antihypertensive medication, *n* (%)	
Yes	10 (13.51)
No	64 (86.49)
Blood pressure at admission	
Systolic pressure (mm Hg)	152.82 ± 31.91
Diastolic pressure (mm Hg) D‐dimer at admission (mg/L) < 0.50 ≥ 0.50	85.84 ± 18.29 30 (40.54) 44 (59.46)
Hemorrhage in the basal ganglia, *n* (%)	34 (45.95)
Left	40 (54.05)
Right	
Bleeding site, *n* (%)	
Mainly located in the lateral aspect of the internal capsule	67 (90.54)
Mainly located in the internal capsule and its medial aspect	7 (9.46)
Hemorrhagic volume (mL), *n* (%)	
≤ 102	64 (86.49)
> 102	10 (13.51)
Brain herniation, *n* (%)	
No	54 (72.97)
Yes	20 (27.03)
90‐day prognosis, *n* (%)	
Good	51 (68.92)
Poor	23 (31.08)

Abbreviation: GCS, Glasgow Coma Scale.

### 2.2 Postoperative Evaluation and Monitoring

The outcomes for patients with PBGCH who were treated with MKCHR were evaluated postoperatively, prior to discharge, and at 30 and 90 days following the procedure through outpatient visits or telephone consultations. The prognosis of the patients was assessed using the modified Rankin Scale (mRS). An mRS score of 0–3 indicated a favorable outcome, while a score of 4–6 denoted an unfavorable outcome.

### 2.3 Statistical Analysis

Statistical analysis was conducted using R version 4.2.0 (http://www.R‐project.org). Data were presented as mean ± standard deviation, percentage, odds ratio (OR), and 95% confidence interval (CI). Risk factors associated with adverse outcomes, as well as their ORs, were identified through univariate and multivariate logistic regression analyses. An online dynamic nomogram was developed based on these independent risk factors using R version 4.2.0 (http://www.Rproject.org) and transformed into a web‐based tool at https://www.shinyapps.io for user convenience. Model performance was assessed through discrimination and calibration measures, with the consistency index (*C*‐index) and receiver operating characteristic (ROC) utilized. Internal validation was conducted via bootstrap resampling, and a calibration curve was generated to depict the relationship between actual and predicted probabilities. The Hosmer–Lemeshow goodness‐of‐fit test's *p*‐value was displayed on the calibration curve to enhance model evaluation rigor and objectivity. Decision curve analysis (DCA), clinical impact curve (CIC), and net reduction interventions (NRI) were used to assess the model's clinical applicability. Statistical significance was set at *p* < 0.05.

## 3 Results

### 3.1 The Characteristics of 74 PBGCH Patients

**FIGURE 1:**
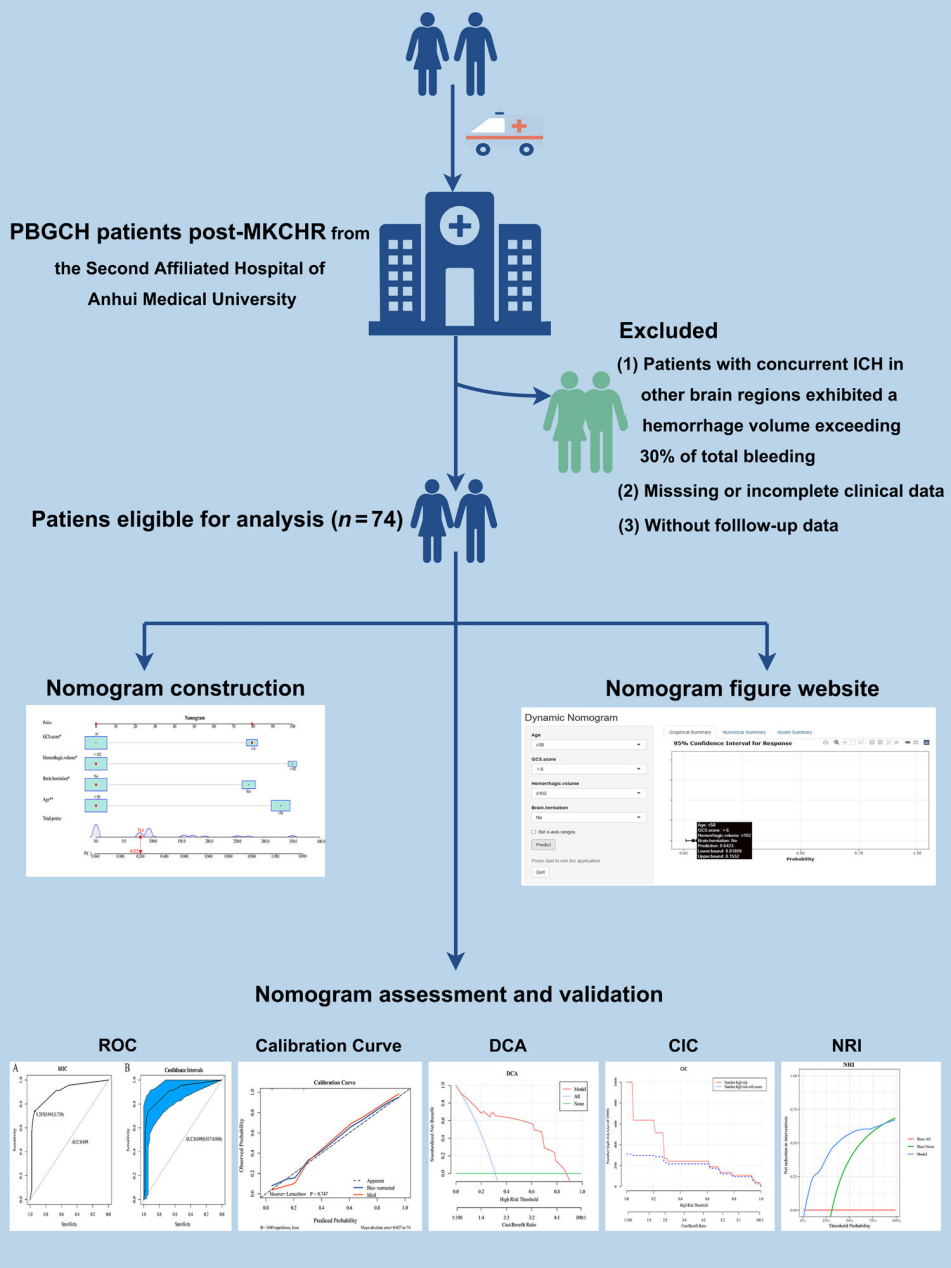
Flowchart of the study design (by Figdraw.com). CIC, clinical impact curve; DCA, decision curve analysis; ICH, intracerebral hemorrhage; MKCHR, microscopic keyhole craniotomy for hematoma removal; NRI, net reduction interventions; PBGCH, patients with primary basal ganglia cerebral hemorrhage; ROC, receiver operating characteristic.

Figure 1 illustrates the patient selection process. The research involved 74 participants who met the inclusion criteria and underwent follow‐up assessments. Patients ranged in age from 27 to 78 years, with 58.11% (*n* = 43) being aged 58 years and above. Most of the patients were male, accounting for 71.62% (*n* = 53), 52.70% (*n* = 39) of junior high school or above, 54.05% (*n* = 40) of urban residents. Most of the 74 patients had a history of smoking or drinking, accounting for 66.22% (*n* = 40). The patients with a history of ischemic stroke accounted for 47.30% (*n* = 35). Those with hyperglycemia made up 52.70% (*n* = 39), while the number of patients with hyperlipidemia was lower, at 17.57% (*n* = 13). All enrolled patients had a history of hypertension; however, only 13.51% (*n* = 10) were taking antihypertensive drugs regularly. Additionally, 58.11% (*n* = 43) sought medical attention within 3 h of symptom onset, and 79.73% (*n* = 59) had a GCS score greater than 6 upon admission. The average systolic blood pressure at admission was 152.82 mm Hg, while the average diastolic blood pressure was 75.85 mm Hg. Additionally, 59.46% (*n* = 44) of patients exhibited abnormal D‐dimer levels upon admission. Furthermore, 72.97% (*n* = 54) of the patients did not experience brain herniation. The bleeding volume in 86.49% (*n* = 64) patients did not exceed 102 mL, and the bleeding in the right basal ganglia was the most common, accounting for 54.05% (*n* = 40). The bleeding sites were mostly outside the internal capsule, accounting for 90.54% (*n* = 67). At 90 days after MKCHR, 68.92% (*n* = 51) of patients had a good prognosis (Table 1).

### 3.2 Establishment of the Nomogram

**FIGURE 2:**
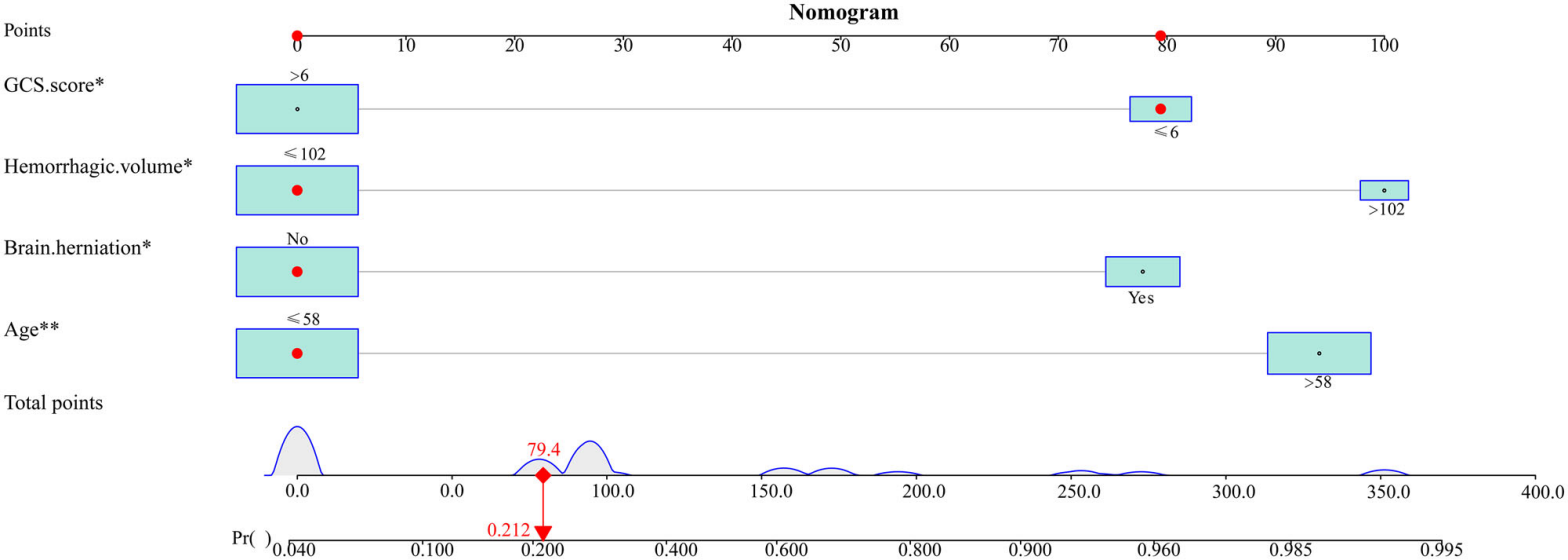
Nomogram was used to predict the 90‐day prognosis of patients with primary basal ganglia cerebral hemorrhage after microscopic keyhole craniotomy for hematoma removal. The Norman chart combines the following four parameters: Glasgow Coma Scale (GCS) score (> 6; ≤ 6), hemorrhagic volume (≤ 102 mL; > 102 mL), brain herniation (No; Yes), age (≤ 58 years; > 58 years). The red arrows indicate the probability of poor prognosis 90 days after surgery in patients with GCS score ≤ 6, hemorrhagic volume ≤ 102 mL, no brain herniation, and age ≤ 58 years. *p < 0.05, **p < 0.01 (statistically significant variables in multivariate logistic regression analysis). Pr, prediction.

**FIGURE 3:**
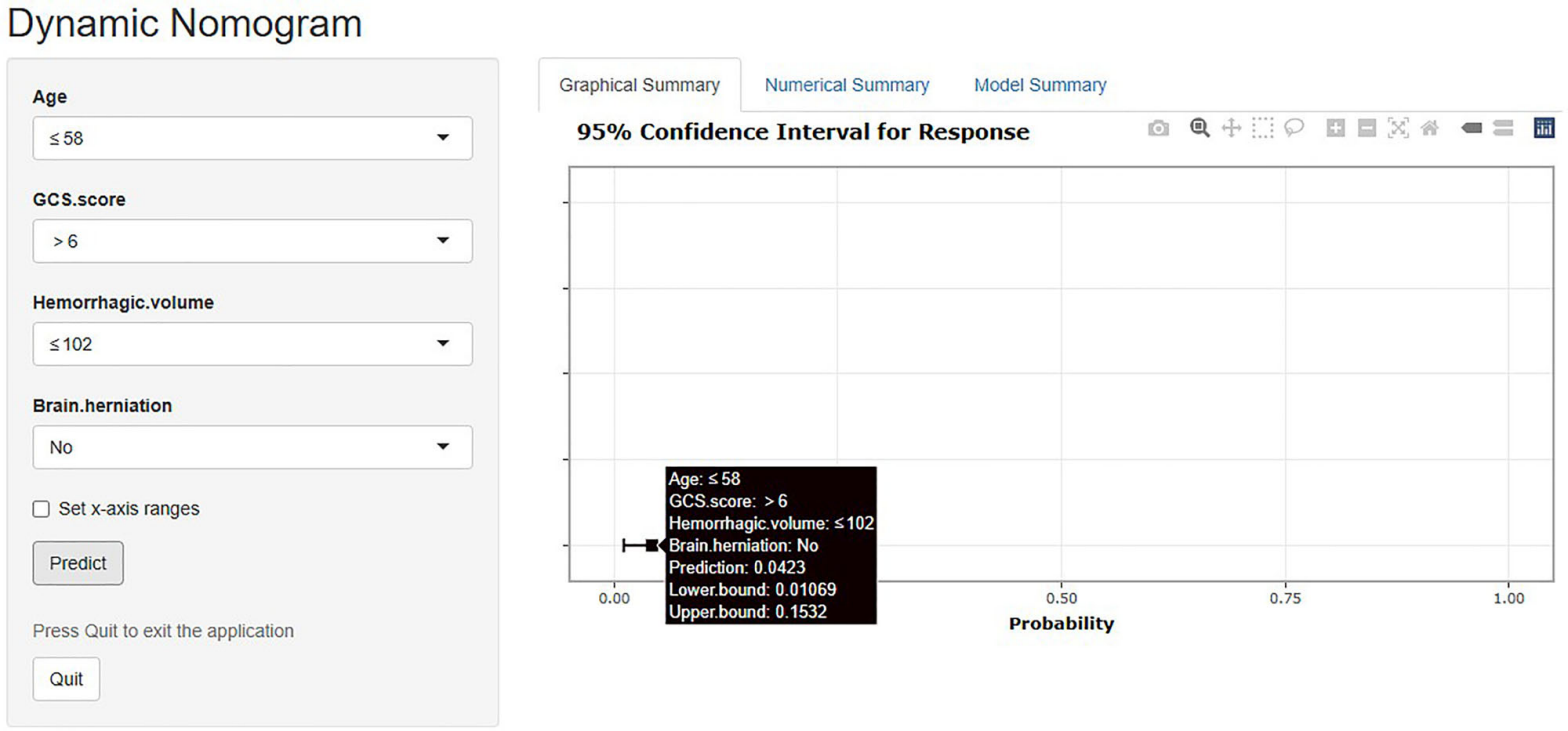
Dynamic nomogram of the probability of poor prognosis 90 days after microscopic keyhole craniotomy for hematoma removal in patients with primary basal ganglia cerebral hemorrhage. To predict the probability of poor prognosis 90 days after microscopic keyhole craniotomy for hematoma removal in patients with primary basal ganglia cerebral hemorrhage with Glasgow Coma Scale (GCS) score > 6, hemorrhagic volume ≤ 102 mL, no brain herniation and age ≤ 58 years.

**FIGURE 4:**
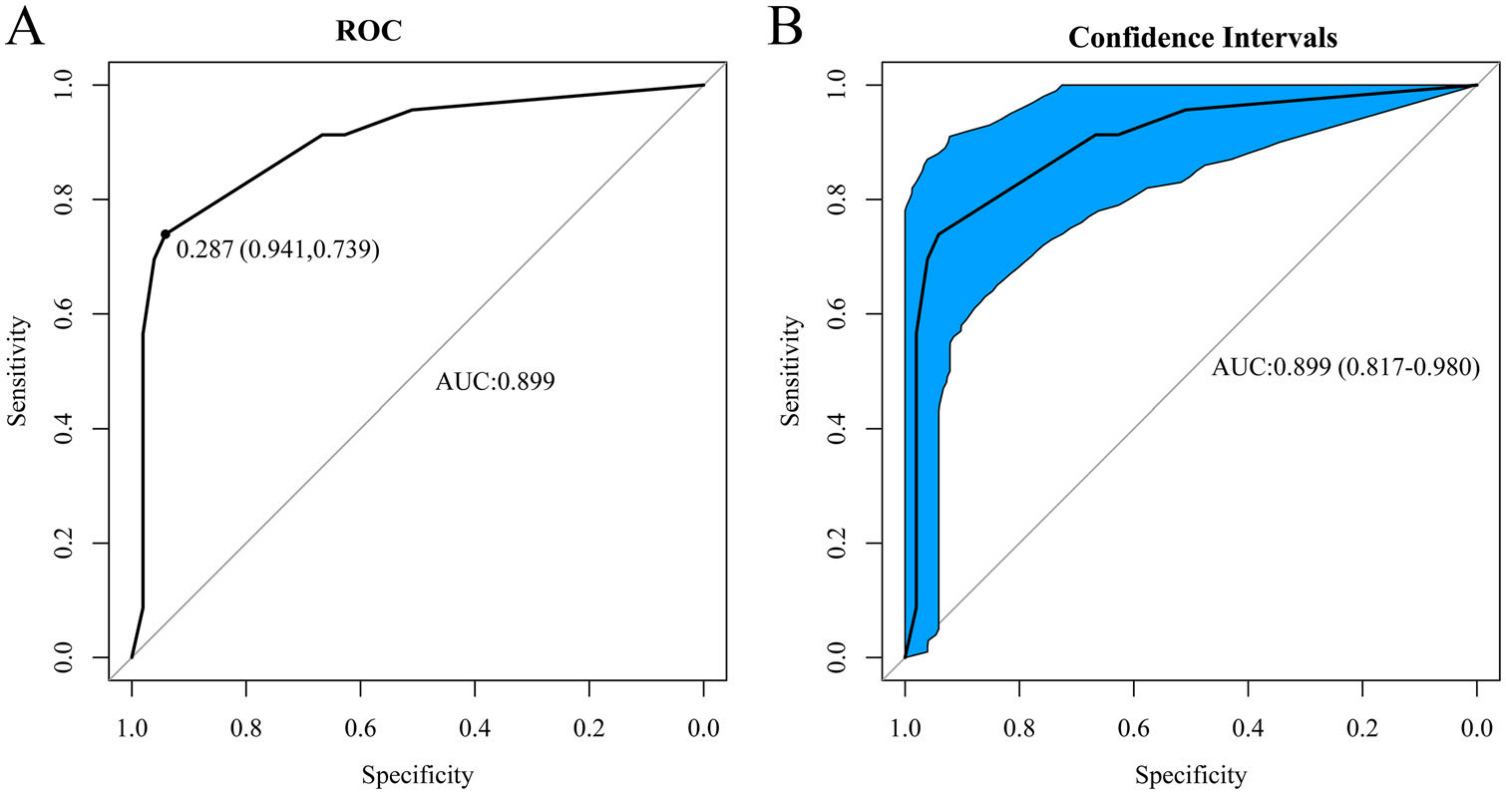
ROC. (A) ROC curve evaluation of discrimination. (B) AUC confidence interval. AUC, area under curve; ROC, receiver operating characteristic.

Univariate and multivariate logistic regression analysis showed that a GCS score ≤ 6 (OR = 6.092, 95% CI, 1.124–33.020, *p* = 0.036), hemorrhagic volume > 102 mL (OR = 9.727, 95% CI, 1.088–86.969, *p* = 0.042), brain herniation (OR = 5.867, 95% CI, 1.130–30.455, *p* = 0.035), and age > 58 years old (OR = 8.488, 95% CI, 1.909–37.745, *p* = 0.005) are independent risk factors for poor prognosis of patients after MKCHR (Table 2). Based on these results, we utilized R software version 4.2.0 to construct a dynamic nomogram (Figure 2) for scoring each clinical parameter. The total score provides an estimation of the likelihood of poor postoperative prognosis. The dynamic Norman figure can be accessed at the website at https://sjwkalg.shinyapps.io/DynNomapp/. Upon completion of the left panel parameters setting, the predicted probability of poor postoperative outcomes for patients is displayed in the right panel (Figure 3). The model demonstrated strong discriminative ability, with an area under the ROC curve of 0.899 (95% CI, 0.817–0.980). At an optimal cut‐off value of 0.287 for the model score, the sensitivity was 0.941, and the specificity was 0.739 (Figure 4). Furthermore, the model exhibited a *C*‐index of 0.899 (95% CI, 0.817–0.980).

**TABLE 2:** The results of the univariate and multivariate logistic regression analysis.

	Univariate analysis			Multivariate analysis		
Characteristics	OR (95% CI)		*p* value	OR (95% CI)		*p* value
Age (years)						
≤ 58	Reference			Reference		
> 58	5.486 (1.876–16.044)		0.002	8.488 (1.909–37.745)		0.005
Gender						
Male	Reference					
Female	1.559 (0.538–4.519)		0.414			
History of smoking or alcohol use						
No	Reference					
Yes	0.711 (0.255–1.982)		0.515			
Junior high school and beyond						
Yes	Reference					
No	1.031 (0.385–2.764)		0.951			
Residence						
City	Reference					
Countryside	0.865 (0.321–2.331)		0.775			
Time from onset to admission (h)						
≤ 3 h	Reference					
> 3 h	0.847 (0.310–2.313)		0.747			
GCS score						
> 6	Reference			Reference		
≤ 6	10.771 (2.911–39.848)		< 0.001	6.092 (1.124–33.020)		0.036
History of ischemic stroke						
No	Reference					
Yes	1.328 (0.495–3.562)		0.573			
Diabetes						
No	Reference					
Yes	2.783 (0.978–7.916)		0.055			
Hyperlipidemia						
No	Reference					
Yes	0.633 (0.160–2.503)		0.515			
Regular intake of antihypertensive medication						
Yes	Reference					
No	0.615 (0.152–2.485)		0.495			
Blood pressure at admission						
Systolic pressure (mm Hg)	0.995 (0.979–1.011)		0.525			
Diastolic pressure (mm Hg) D‐dimer at admission (mg/L) < 0.50 ≥ 0.50	0.982 (0.954–1.010) Reference 1.878 (0.660–5.341)		0.210 0.238			
Hemorrhage in the basal ganglia						
Left	Reference					
Right	0.865 (0.321–2.331)		0.775			
Bleeding site						
Mainly located in the lateral aspect of the internal capsule	Reference					
Mainly located in the internal capsule and its medial aspect	3.368 (0.688–16.492)		0.134			
Hemorrhagic volume (mL)						
≤ 102	Reference			Reference		
> 102	13.067 (2.500–68.304)		0.002	9.727 (1.088–86.996)		0.042
Brain herniation						
No	Reference			Reference		
Yes	5.864 (1.927–17.847)		0.002	5.867 (1.130–30.455)		0.035

Abbreviations: GCS, Glasgow Coma Scale; OR, odds ratio; 95% CI, 95% confidence interval.

### 3.3 Nomogram Validation

**FIGURE 5:**
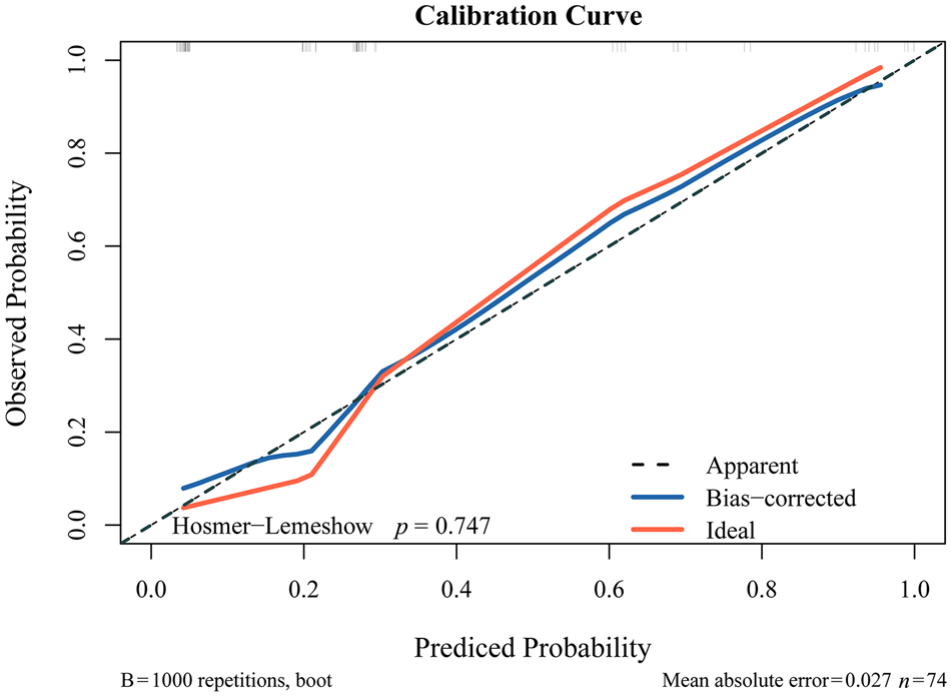
Calibration curve of nomogram. The *X*‐axis represents the probability of prediction and the *Y*‐axis represents the probability of observation. The black dashed line indicates a perfect prediction. The solid red line represents the entire queue (*n* = 74), and the solid blue line was corrected for deviation through bootstrap (1000 repetitions) to show the observed Norman graph performance.

**FIGURE 6:**
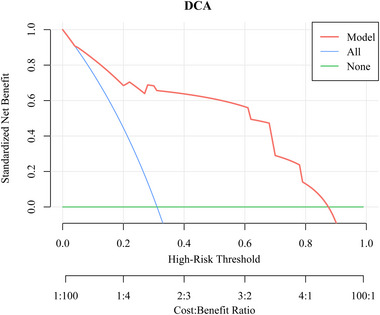
Decision curve analysis of nomogram. When the high‐risk threshold probability was 0%–88%, the model achieved a greater net benefit than patients who had all or no surgery. DCA, decision curve analysis.

**FIGURE 7:**
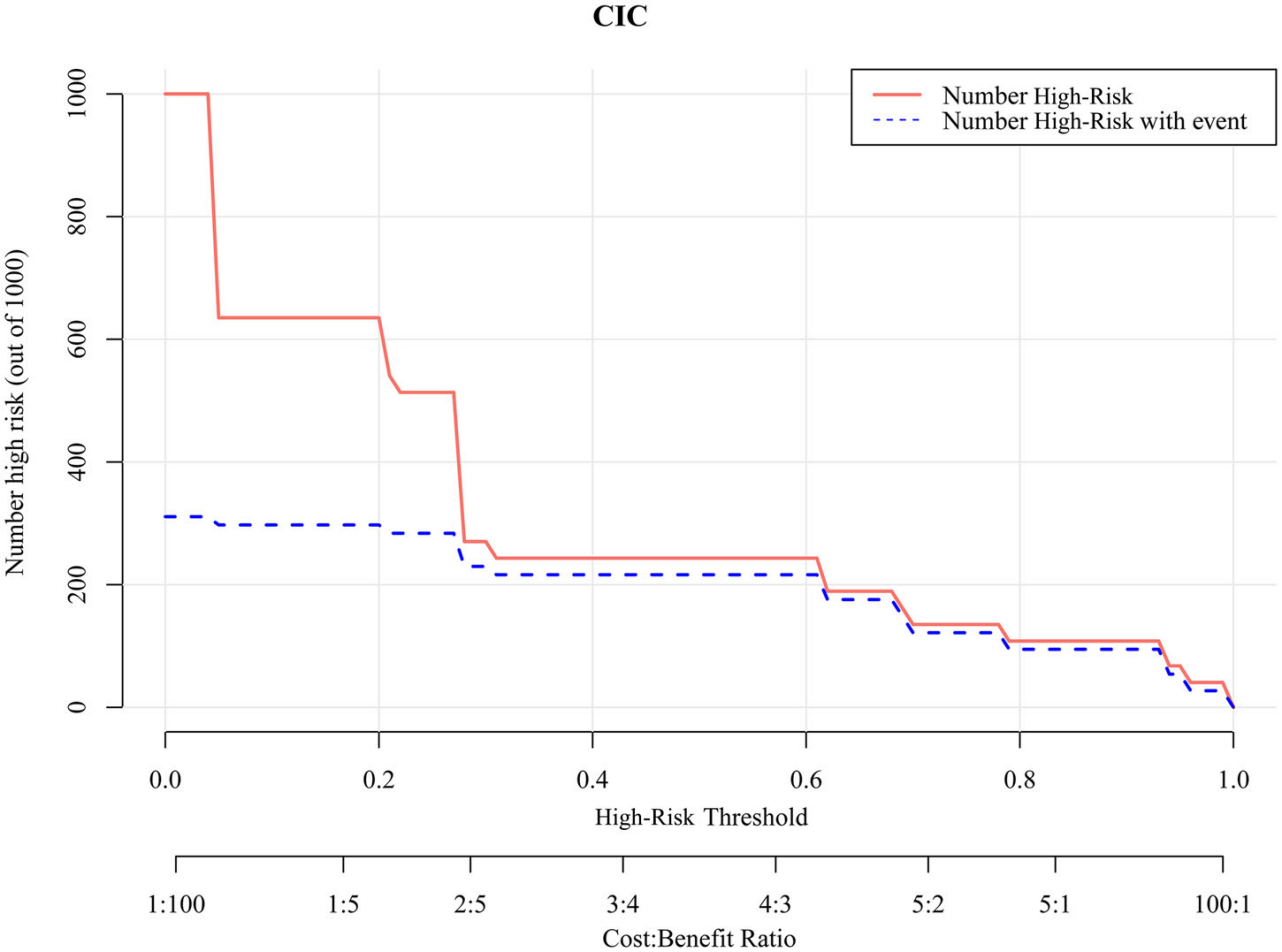
Clinical impact curve of nomogram. The estimated number of patients at high risk for each potential risk threshold is shown, and the proportion of patients with true positive cases is visualized. CIC, clinical impact curve.

**FIGURE 8:**
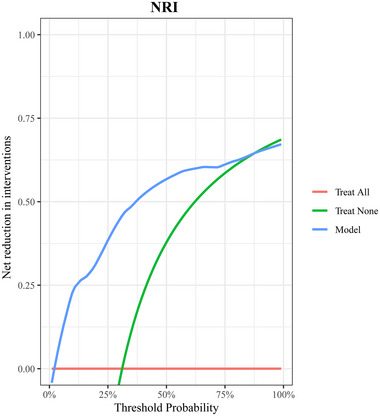
Net reduction interventions of nomogram. The net reduction in intervention for each potential risk threshold is shown. NRI, net reduction interventions.

Bootstrap resampling was employed for internal validation of the model, yielding a *C*‐index of 0.90. A calibration curve (Figure 5) indicated that the predicted and actual probabilities of poor prognosis closely aligned along the Y = X line. The Hosmer–Lemeshow goodness‐of‐fit test showed *p* > 0.05, signifying well‐calibrated model performance. In addition, DCA depicted in Figure 6 illustrates the clinical utility of the model. It indicates that the model can offer a greater net benefit compared to scenarios where all patients undergo surgery or no surgery when the probability of the high‐risk threshold falls within the range of 0%–88%. CIC in Figure 7 displays the estimated count of high‐risk patients at various risk thresholds and illustrates the proportion of patients with true positive cases. At a 40% risk threshold, approximately 220 out of 1000 screened patients would be considered high risk, and about 210 of those patients would be truly poor postoperative. NRI (Figure 8) shows the net reduction in interventions for each potential risk threshold. For example, when the risk threshold is 37.5%, the net reduction in interventions using the model is about 50 interventions per 100 patients. Notably, the net reduction in interventions using the model consistently surpasses that of no surgery or all surgery when the probability is between 0% and 88%.

## 4 Discussion

With the advancement of minimally invasive techniques, microscopic keyhole or microbone window craniotomy for hematoma has been increasingly adopted by medical units throughout China for the treatment of PBGCH. This study is the first to identify short‐term prognostic risk factors in PBGCH patients who were treated with MKCHR and to develop an online prediction model. Similar to previous studies (Akpinar et al. 2019; Lin et al. 2023; Zou et al. 2022), we found that age > 58 years, a GCS score ≤ 6, hemorrhagic volume > 102 mL, and brain herniation were independent risk factors for 90‐day postoperative prognosis. In this study, we established the first dynamic model for predicting the prognosis of patients with PBGCH treated by MKCHR.

Jenett and Bond first developed the GCS in the 1970s, when it was used to assess the severity of head injuries and guide treatment. Later studies have further shown that “GCS” score is an important evaluation index of neuronal injury, and can be widely used to evaluate the condition and prognosis of various types of brain damage. It assesses the patient's response in three aspects: eye opening, verbal response, and motor response. The lower the score, the more serious the degree of nerve damage (Babi and James 2017). Studies by Babi et al. have shown that GCS score is a key risk factor affecting a patient's prognosis after cerebral hemorrhage (An et al. 2017; Parry‐Jones et al. 2013). In our study, we found that patients with a GCS > 6 at admission were more likely to have a better prognosis after minimally invasive hematoma removal under the microscope through keyhole craniotomy.

Among patients experiencing cerebral hemorrhages, the hemorrhagic volume is widely recognized as an independent risk factor influencing short‐term outcomes (Gregório et al. 2018). This is because the mechanical destruction of hematoma not only leads to the primary damage to the basal ganglia but also causes secondary damage. For example, after the formation of hematoma, thrombin, iron ions, and hemoglobin produced by the degradation of red blood cells can trigger abnormal reactions in the coagulation mechanism, inflammatory reactions, toxic reactions to neurons, and other pathological processes, which further aggravate the damage to brain tissue. This increases the risk of blood vessel damage, brain edema, and neuronal cell death. At present, it is widely accepted in the field of neurosurgery that minimally invasive surgery can be used to treat patients with primary cerebral hemorrhage when the hematoma volume is between 30 and 80 mL, as it can reduce surgical trauma and achieve good results. Our study further confirms this view, revealing that in some patients, hematoma volumes exceeding 80 mL—up to 102 mL—can still lead better prognosis. This finding provides a reference for the treatment of patients with hematoma volumes between 80 and 102 mL, suggesting that microinvasive hematoma removal through keyhole craniotomy may be effective even in cases of larger blood loss. Clinically, cerebral hemorrhage is sometimes accompanied by the formation of cerebral herniation. Therefore, we included patients with brain herniation in the study and found that it can be an independent risk factor for the prognosis of patients with PBGCH undergoing minimally invasive hematoma removal under the microscope via keyhole craniotomy. This finding is consistent with results reported in previous studies, which indicate that cerebral herniation in patients prior to surgery is an independent risk factor for poor prognosis in those with traumatic brain injury (Chen et al. 2024; Shen et al. 2023).

Age has been identified as a significant risk factor for acute cerebral hemorrhage, with its incidence rising as individuals age (van Asch et al. 2010). Aging is often associated with underlying conditions such as diabetes and cardiovascular disease, all of which contribute to varying degrees of poor prognosis in cerebral hemorrhage outcomes. Quality of life poststroke typically declines with age (Sprigg et al. 2013). There is evidence that older stroke patients are more likely to develop depression than their younger counterparts (Lincoln et al. 2013). Symptoms of depression and diminished social support contribute to poorer health outcomes, even when accounting for physical functioning and treatment following a major stroke. In a study by Radholm et al., advanced age was strongly linked to death and severe disability within 90 days following cerebral hemorrhage (Radholm et al. 2015). According to our study, patients > 58 years of age had an 8.488‐fold higher risk of poor prognoses after surgery than those ≤ 58 years of age.

In recent years, clinical prediction models have been widely adopted by medical professionals for the prognostic analysis of patients with conditions such as brain injury and various cancers, gradually replacing traditional prediction models (Liu et al. 2024; Miao et al. 2024; Zhuang et al. 2024). In the past, there were a few prognostic models about PBGCH, all of which believed that the prediction model established by multiple independent risk factors could predict the prognosis of patients with PBGCH after surgery, thus reducing the incidence of clinical unnecessary surgery and providing theoretical decision‐making reference for clinicians and patients before surgery. However, most of the previous prediction models were limited to the establishment of multifactor independent risk factor models, or the effects of different surgical procedures were not considered when the models were established. There was no short‐term prognosis prediction model specifically for patients with primary basal ganglia hemorrhage undergoing minimally invasive hematoma removal through keyhole craniotomy under the microscope, or only a static prognosis scoring system was provided. And other clinicians or patients cannot use it directly, it is not convenient to use (Baker et al. 2024; Wu et al. 2023; Zhang et al. 2022; Zyck et al. 2020). In this study, the R software was utilized to develop a user‐friendly web‐based version of the prediction model, offering enhanced convenience and precise data visualization. As illustrated in Figure 3, the nomogram is accessible at the following URL: https://sjwkalg.shinyapps.io/DynNomapp/. By inputting personalized information for individual patients, the system identifies specific independent risk factors linked to prognosis and provides real‐time prediction probabilities. For example, for patients with a GCS score > 6, hemorrhagic volume ≤ 102 mL, age ≤ 58 years, and no brain herniation, the predictive value of this system is 0.0423, and the incidence of adverse prognosis is recommended for surgical treatment. It is easy to use, and the information is accurate and easy to understand.

There are some limitations to the study. First, this was a single‐center study, and differences in the level of surgery at different hospitals may impact patient outcomes. Subsequent research will aim to increase the sample size at this center to enhance model accuracy, alongside conducting multicenter studies for external validation. Second, the retrospective nature of the study introduces susceptibility to selection bias, as the patient cohort may not be representative of the broader population. This bias can arise from factors such as the criteria used for patient inclusion, the decision‐making processes of physicians, and the availability of resources. Acknowledging these factors is essential, as they may affect the generalizability of our findings. In addition, it is crucial for physicians to consider various factors such as the patient's general health, comorbidities, preferences, and predictive model outputs when devising an appropriate treatment plan. Finally, this cohort was followed for 90 days, but a longer follow‐up period is to adequately test the prognostic risk model.

## 5 Conclusion

In summary, this study found that, upon admission, patients with PBGCH who had a GCS score ≤ 6, hemorrhagic volume > 102 mL, brain herniation, and age > 58 years had a significantly increased risk of poor prognosis following MKCHR. The online dynamic nomogram constructed by the above variables can predict the possibility of poor short‐term prognosis of patients after microinvasive hematoma removal via the keyhole approach. ROC, calibration curve, DCA, CIC, and NRI show that nomogram has predictive accuracy, calibration performance, and clinical applicability. This model effectively differentiates the outcomes of patients with PBGCH undergoing MKCHR, thereby reducing the number of unnecessary procedures. This assessment system can assist in selecting clinical surgical treatment plans and evaluating postoperative prognosis for patients with PBGCH.

## Author Contributions

**Hongliang Wang**: conceptualization, methodology, data curation, investigation, writing–original draft, writing–review and editing. **Sai Li**: methodology, software, formal analysis, writing–original draft, writing–review and editing. **Yang Nie**: data curation, investigation, formal analysis. **Chenxi Chang**: investigation, formal analysis. **Haoyuan Wu**: investigation, formal analysis. **Bing Zhao**: conceptualization, supervision. All authors read and approved the final manuscript.

## Ethics Statement

This study was conducted by the Ethics Committee of the Second Affiliated Hospital of Anhui Medical University (Hefei; China; approval no. 20240102). The Declaration of Helsinki was followed. All patients provided written informed consent.

## Consent

All patients provided written informed consent.

## Conflicts of Interest

The authors declare no conflicts of interest.

### Peer Review

The peer review history for this article is available at https://publons.com/publon/10.1002/brb3.70344

## Data Availability

The datasets used and/or analyzed during the current study are available from the corresponding author on reasonable request.
